# High-Grade Small Bowel Dysplasia Following Prior Adenocarcinoma in Small Bowel Crohn’s Disease

**DOI:** 10.7759/cureus.106330

**Published:** 2026-04-02

**Authors:** Farhan S Mohiuddin, Laine Lyles, Nisha Loganantharaj

**Affiliations:** 1 Internal Medicine, Louisiana State University Health Sciences Center, New Orleans, USA; 2 Gastroenterology, Louisiana State University Health Sciences Center, New Orleans, USA

**Keywords:** crohn's disease, endoscopic screening, inflammatory bowel disease (ibd), small bowel malignancy, small bowel resection

## Abstract

Patients with Crohn’s disease are at increased risk of small bowel adenocarcinoma due to chronic inflammation, though absolute incidence is low and surveillance guidelines are unclear. We report a patient with long-standing small bowel Crohn’s disease and prior adenocarcinoma who developed high-grade dysplasia despite clinical, biochemical, and endoscopic remission on biologic therapy. An asymptomatic rise in fecal calprotectin prompted imaging and device-assisted enteroscopy, revealing a non-traversable stricture. Surgical resection confirmed high-grade dysplasia with negative margins. This case underscores persistent malignancy risk in fibrostenosing Crohn’s disease and the importance of biomarkers and advanced diagnostics in high-risk patients.

## Introduction

Malignant neoplasms, primarily consisting of small bowel adenocarcinomas (SBA) and neuroendocrine tumors, arising from the small intestines are rare, with 22.7 cases per million people in the United States reported in 2004 [1,2]. In the general population, the lifetime risk of developing SBA was reported to be 0.3% in 2015 [3]. Crohn’s disease (CD) results in the release of proinflammatory mediators, causing chronic inflammatory changes that involve the gastrointestinal tract and increase the risk of carcinogenesis [4]. Patients with CD have an increased risk of developing SBA, with an incidence of 0.3/100 patient-years being reported [5]. We present a CD case showing a patient with high-grade small bowel dysplasia with prior SBA on advanced therapy.

## Case presentation

A 75-year-old man with jejunal and ileocolonic Crohn’s disease presented to an academic inflammatory bowel disease center to establish care. At the time of diagnosis, when he was 20 years old, his gastrointestinal symptoms included chronic diarrhea, abdominal pain, and weight loss. His disease course was complicated by multiple jejunal and ileal strictures, requiring an ileocectomy in 1971 and multiple small bowel resections in 2007. He started on oral corticosteroids in 1974 and continued them daily for approximately 33 years. In 2007, he underwent resection of multiple small bowel strictures, including a jejunal stricture complicated by a jejuno-jejunal fistula. Surgical pathology of the jejunal stricture was significant for small bowel, moderately differentiated invasive adenocarcinoma. Postoperatively, he was started on mercaptopurine (6-MP) from 2007 to 2021. Clinically, he continued to have diarrhea and abdominal pain; however, symptoms were less severe than at the time of diagnosis. He had multiple squamous cell skin cancers requiring Mohs resection; therefore, 6-MP was stopped in 2021, and he was started on oral budesonide. He had worsening abdominal cramping, diarrhea, and a 20 lb weight loss. CT abdomen was significant for mural thickening and hyperenhancement within the distal small bowel. He was started on prednisone 20 mg but was unable to taper due to a recurrence of symptoms. Prior genetic testing was performed with no detection of Lynch syndrome, familial adenomatous polyposis (FAP), Peutz-Jeghers syndrome, or serrated polyposis syndrome.

At the time of establishing care with a new provider, he denied abdominal pain, had 2-3 formed bowel movements, and had weight gain while on prednisone 20 mg daily. Restaging of the disease with esophagogastroduodenoscopy (EGD) showed patchy linear ulcerations and aphthous ulcers in the mid-to-distal esophagus and duodenal erosions. Colonoscopy was significant for a normal colon with ileocolonic side-to-side anastomosis and normal neo-terminal ileum (Rutgeerts score i0). He was tapered off prednisone and started on infliximab monotherapy with therapeutic drug monitoring to manage his Crohn’s disease. He was dose-optimized based on proactive therapeutic drug monitoring to 10 mg/kg every 4 weeks. He achieved clinical and endoscopic remission on serial endoscopies as well as normalization of his fecal calprotectin.

**Figure 1:**
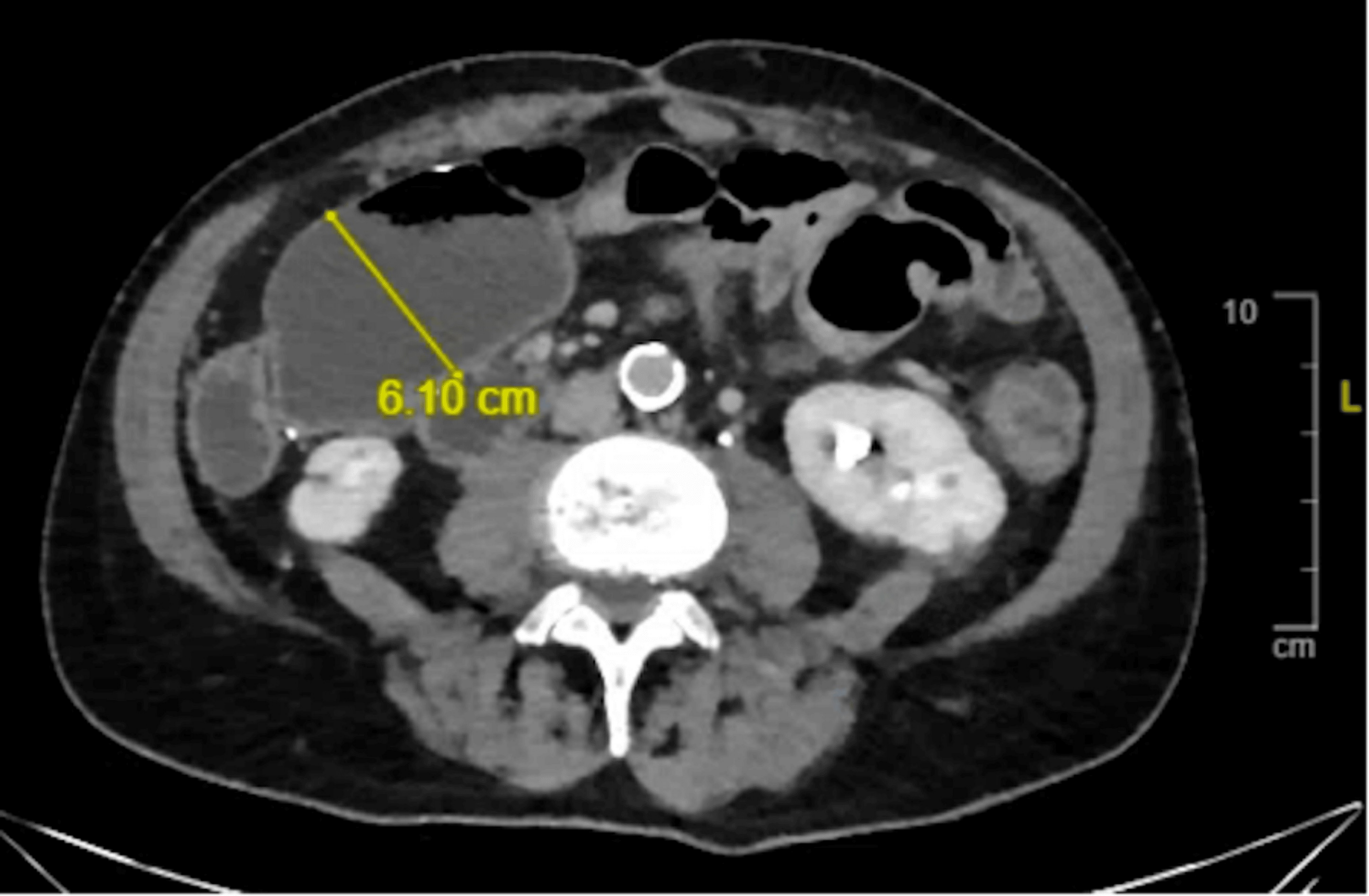
CT showing circumferential transmural wall thickening of the distal ileum with skip lesions

**Figure 2:**
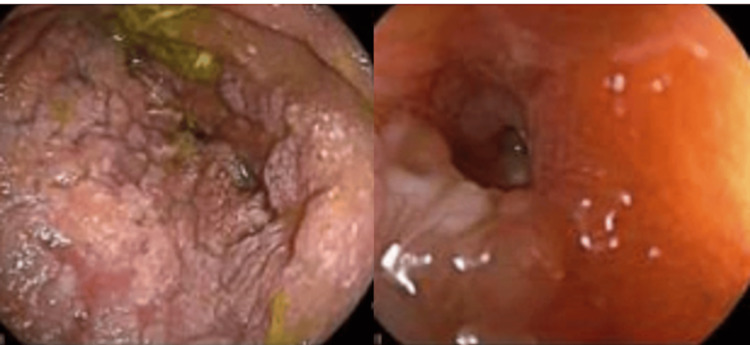
Image captured on double balloon endoscopy of the small bowel stricture

Several years later, fecal calprotectin was elevated on routine evaluation. The patient clinically felt well and was adherent to the therapeutic dose of infliximab monotherapy. CT enterography (CTE) was performed and was significant for fibrostenosing disease with multiple skip lesions, primarily affecting the jejunum and distal ileum, with upstream dilation of 6.1 cm (Figure 1). Double-balloon enteroscopy revealed a severe stricture in the distal ileum/proximal jejunum that could not be transversed with the enteroscope (Figure 2). A tattoo was placed proximal to the stricture, and he was referred to colorectal surgery. Jejunal resection with side-to-side functional end anastomosis was performed; surgical pathology was significant for active Crohn’s enteritis of the jejunum, high-grade dysplasia with clear margins, and enteritis cystica profunda.

## Discussion

Patients with CD are at an increased risk of dysplasia and adenocarcinoma as compared with the general population, although the absolute incidence remains low. Population-based cohort studies and meta-analyses have demonstrated a 6-to-18-fold increased risk of SBA in CD, with an emphasis on those with long-standing disease and small bowel involvement [6-8]. Those with stricturing or penetrating disease phenotypes are associated with the highest risk of the development of malignancy [9-11]. The risk of malignancy increases with disease duration exceeding 8-15 years, advanced age (50 years or older), ileal involvement, and childhood-onset CD, which suggests a cumulative inflammatory burden as a driver of carcinogenesis [12,13]. Chronic inflammation is thought to promote neoplastic transformation through an inflammation-dysplasia-adenocarcinoma sequence [14,15]. In this manuscript, fecal calprotectin served as a noninvasive biomarker of intestinal inflammation and assisted in identifying ongoing subclinical inflammatory activity in patients who appear clinically quiescent [16]. Strictures may harbor occult malignancy with neoplasia often identified incidentally at the time of surgical resection for presumed fibrostenosing disease [17]. There are no current guidelines established regarding screening with small bowel endoscopy in this patient population during remission, including patients with prior small bowel pathology.

## Conclusions

The diagnosis of SBA in CD remains challenging due to nonspecific symptoms, overlap with inflammatory or obstructive manifestations of the disease process, and limited access to small bowel endoscopic evaluation, resulting in delayed detection. Despite these recognized risks, there are no established surveillance guidelines for SBA in patients with CD. It is acknowledged in practice guidelines that there is an increased malignancy risk, but the role of routine surveillance has not been determined, especially for those with prior dysplasia. This case highlights the importance of using imaging and device-assisted enteroscopy to evaluate small bowel Crohn’s disease patients who are at high risk for recurrent small bowel adenocarcinoma despite clinical symptoms.
